# Analysis of somatic copy number alterations in biliary tract carcinoma using a single nucleotide polymorphism array

**DOI:** 10.2144/fsoa-2021-0057

**Published:** 2021-11-15

**Authors:** Yoshihiro Shioi, Mitsumasa Osakabe, Naoki Yanagawa, Hiroyuki Nitta, Akira Sasaki, Tamotsu Sugai

**Affiliations:** 1 Department of Molecular Diagnostic Pathology, School of Medicine, Iwate Medical University, 2-1-1, Shiwagun, Yahabachou, 0283695, Japan; 2 Department of Surgery, School of Medicine, Iwate Medical University, 2-1-1, Shiwagun, Yahabachou, 0283695, Japan

**Keywords:** bile duct cancer, biliary tract carcinoma, carcinogenesis, cluster analysis, crypt isolation method, gall bladder cancer, molecular alteration, prognosis, single nucleotide polymorphism array, somatic copy number alteration

## Abstract

**Aim:**

Biliary tract carcinoma (BTC), including gall bladder carcinoma (GBC) and biliary duct carcinoma (BDC), has a poor prognosis. Comprehensive genomic profiling has important roles in evaluation of the carcinogenesis of BTC.

**Materials & methods:**

We examined somatic copy number alterations (SCNAs) using a single nucleotide polymorphism array system to analyze 36 BTC samples (11 GBCs and 25 BDCs).

**Results:**

In hierarchical cluster analysis, two clusters were identified (subgroup 1 with low SCNAs and subgroup 2 with high SCNAs). GBC was predominant in subgroup 1, whereas BDC was predominant in subgroup 2, suggesting that GBC and BDC had different genetic backgrounds in terms of SCNAs.

**Conclusion:**

These findings could be helpful for establishing the molecular carcinogenesis of BTCs.

## Lay abstract

Biliary tract carcinoma, including gall bladder carcinoma (GBC) and biliary duct carcinoma (BDC), has a poor prognosis. Comprehensive genomic (single nucleotide polymorphism-array) profiling plays important roles in evaluation of the carcinogenesis of biliary tract carcinoma. In the hierarchical cluster analysis, two clusters were identified (subgroup 1 with low somatic copy number alterations [SCNAs] and subgroup 2 with high SCNAs); GBC was found to be predominant in subgroup 1, whereas BDC was predominant in subgroup 2. These findings suggested that GBC and BDC had different genetic backgrounds in terms of SCNAs.

Biliary tract carcinoma (BTC) includes cholangiocarcinoma (CCC; biliary duct carcinoma [BDC]) and gall bladder carcinoma (GBC); the former is classified into intrahepatic, perihilar and distal subtypes [1]. Although the latter two subtypes were previously grouped as extrahepatic CCC, these subtypes are now considered distinct entities based on differences in their tumor biology and management. Peripheral CCC is the most common subtype [1]. However, GBC is also one of the most common cancers among BTCs. The prognosis of BTC, including BDC and GBC, is considered dismal [1–4]. Early detection and diagnosis of BTC is delayed in routine practice, as supported by the finding that although GBC is suspected pre-operatively in only 30% of all patients, the other 70% of cases are diagnosed using postoperative incidental findings by a pathologist [2]. To improve early detection and diagnosis of BTC, it is important to further improve our understanding of the molecular tumor biology of BTC.

Previous studies have shown that molecular alterations in BTC include mutations in *KRAS*, *TP53*, *SMAD4*, *HER*, *PIK3CA*, *MET* and *IDH2* [5,6]. The profile derived from such molecular analyses identified the activation of pathways driving proliferation (i.e., *RAS*, *PIK3CA*, *HER2* and *MET*) [5,6]. Recently, next-generation sequencing has proven useful for identifying mutations in specific genes occurring in neoplastic cells [6]. Although mutation analysis using next-generation sequencing can provide important findings for evaluating human carcinogenesis [6], mutations alone may not fully predict tumor invasiveness and growth during neoplastic progression. Somatic copy number alterations (SCNAs), which are closely associated with neoplastic progression and potential metastatic ability, have attracted much attention in the field of cancer research [7–13]. Identification of SCNAs has been used in genome-wide analyses of human neoplasias, including gastrointestinal, lung, gynecological and BTCs, providing insights into whole molecular alterations found in human tumors [12,14–17]. In addition, comprehensive genomic profiling of SCNAs may have important roles in guiding systemic, personalized anticancer therapy.

Accordingly, in this study, we aimed to provide important insights into the potential benefits of SCNA profiling in patients with BTC.

## Materials & methods

### Patients

Thirty-six cases of surgically resected BTC, including 11 GBCs and 25 BDCs, were evaluated in this study. Medical records were available and carefully reviewed. Patients with pretreatment, including chemotherapy and radiotherapy, were not included. Pathological diagnoses and descriptions of clinicopathological variables were made in accordance with the Histological Classification of General Rules for Clinical and Pathological Studies on Cancer of the Biliary Tract, with slight modifications [18]. In addition, carcinoma of the ampulla of Vater, derived from the intrapapillary bile duct, included BDC. Clinicopathological findings are shown in Table 1.

**Table 1.** Clinicopathological findings of the biliary tract carcinoma we examined.

	Total (%)	Gallbladder carcinoma (%)	Bile duct carcinoma (%)
Total	36	11 (30.6)	25 (69.4)
**Sex**			
Man	24 (66.7)	5 (20.8)	19 (79.2)
Woman	12 (33.3)	6 (50)	6 (50)
Age (years), median (range)	70 (41–86)	71 (41–84)	70 (45–86)
**Location**			
Gallbladder carcinoma (Gn/Gb/Gf)		2/3/6	
Bile duct carcinoma (A/Bi/Bm/Bs/Bp/C)			4/10/5/2/1/3
Size (mm), median (range)	29 (8–123)	45 (15–123)	27 (8–75)
Histological subtype (pap/well/mod/muc/por)	17/10/6/1/2	7/1/1/0/2	10/9/5/1/0
**Stage**			
I	14 (38.9)	3 (21.4)	11 (78.6)
II	17 (47.2)	4 (23.5)	13 (76.5)
III	2 (5.6)	2 (100)	0 (0)
IV	3 (8.3)	2 (66.7)	1 (33.3)
**Adjuvant chemotherapy**			
Done	13 (36.1)	4 (30.8)	9 (69.2)
None	23 (63.9)	7 (30.4)	16 (69.6)
**Recurrence**			
Presence	21 (58.3)	7 (33.3)	14 (66.7)
Absence	15 (41.7)	4 (26.7)	11 (73.3)
Progression-free survival period (days), median (range)	1208.5 (97–4049)	368 (97–4049)	1250 (111–3952)
**Mortality**			
Death	18 (50)	7 (38.9)	11 (61.1)
Survival	18 (50)	4 (22.2)	14 (77.8)
Overall survival period (days), median (range)	1480 (151–4049)	675 (151–4049)	1624 (227–3952)

A: Ampulla of Vater; Bi: Inferior bile duct; Bm: Middle bile duct; Bp: Perihilar bile duct; Bs: Superior bile duct; C: Cystic duct; Gb: Body of gallbladder; Gf: Fundus of gallbladder; Gn: Neck of gallbladder; mod: Moderately differentiated adenocarcinoma; muc: Mucinous carcinoma; pap: Papillary adenocarcinoma; por: Poorly differentiated adenocarcinoma; well: Well differentiated adenocarcinoma.

### Crypt isolation technique for tumor cell isolation

Fresh tumor and normal tissue samples were obtained from surgical specimens removed during surgery for BTC. The tumor samples were obtained primarily from the central area of the tumor, and included the most invasive layer of the tumor. A sample of normal colonic mucosa was removed from a site distant from the lesion.

Crypt (gland) isolation from tumors (36 cases) and corresponding normal mucosa (36 cases), which were obtained from the superficial epithelium, was performed as previously described [19]. Briefly, fresh tumor and mucosa tissues were minced with a razor into minute pieces and then incubated at 37°C for 30 min in calcium- and magnesium-free Hanks’ balanced salt solution (CMF) containing 30 mM ethylene-diaminetetraacetic acid. Following this procedure, the tissues were then stirred in CMF for 30–40 min. The isolated glands were immediately fixed in 70% ethanol and stored at 4°C until DNA extraction. The fixed isolated glands were examined under a dissection microscope (SZ60, Olympus, Tokyo, Japan).

The isolated glands were routinely processed for histopathological analysis to morphologically confirm their isolated nature. No contamination, such as interstitial cells was observed in any of the 36 samples.

### DNA extraction

Genomic DNAs from isolated cancer glands and normal epithelium were extracted and prepared from the isolated crypts as described previously [19].

### Single nucleotide polymorphism array analysis

The Cytoscan HD platform (Affymetrix, Cheshire, UK) was used in all experiments. This array contains more than 1.9 million nonpolymorphic markers and over 740,000 SNP markers, with an average intragenic marker spacing of 880 bp and intergenic marker spacing of 1737 bp. All procedures were carried out according to the manufacturer’s instructions. The hybridized slides bearing DNA marked with biotin were analyzed with a GeneChip Scanner 3000 7G (Affymetrix) and Chromosome Analysis Suite Software (Affymetrix). The definitions of abnormalities and the detailed methodology were described previously [20].

### Classification of copy number alterations

In the current study, we classified SCNAs into three subtypes, including gain, loss of heterozygosity (LOH), and copy neutral LOH (CN-LOH) [20]. Whereas LOH was defined as a cross-chromosomal change that consequently led to loss of the entire gene and the surrounding region, gain was defined as a cross-chromosomal change that resulted in a gain of the entire gene and the surrounding region. CN-LOH was defined as an occurrence of LOH in the absence of allelic loss (copy number = 2).

### Hierarchical analysis of copy number alterations

We performed hierarchical cluster analysis to separate the samples based on their SCNA patterns. This approach maximized homogeneity for each group and guaranteed the greatest differences between groups. Open-access clustering software (Cluster 3.0 software; bonsai.hgc.jp/∼mdehoon/software/cluster/software.htm) was used for cluster analysis. The clustering algorithm was set to centroid linkage clustering, which is the standard hierarchical clustering method used in biological studies [15,20].

### Statistical analysis

Differences in clinicopathological findings were examined using chi-square tests in Stat Mate-III (Atom, Tokyo, Japan). Findings included sex, tumor size (≤40 and >40 mm), tumor location, tumor node metastasis stage, adjuvant chemotherapy, recurrence and overall survival. Differences in age and tumor size distributions among the groups were analyzed using Kruskal–Wallis H tests in Stat Mate-III. Results with a p-value less than 0.05 were considered significant. Differences in SCNA patterns among subgroups were evaluated with Fisher’s exact tests with an adjusted Bonferroni correction.

We calculated progression-free survival (without metachronous metastasis) of the patients based on the date of surgery and the date of the last follow up or patient metachronous metastasis. In addition, overall survival of the patients was examined. The association was analyzed using Kaplan–Meier analysis. The level of significance was set at p < 0.05. Statistical analyses were conducted with the JMP Pro 13.0 software package (SAS Institute, Inc., NC, USA) for Windows.

## Results

The median total number of chromosomal aberrations per patient was 227 (range: 37–768), with a median of 65 gains (range: 1–457), 135 LOHs (range: 7–373) and 26 CN-LOHs (range: 3–143) in BTC including BDC and GBC.

### Hierarchical clustering based on SCNA patterns in BTC

**Figure 1.**
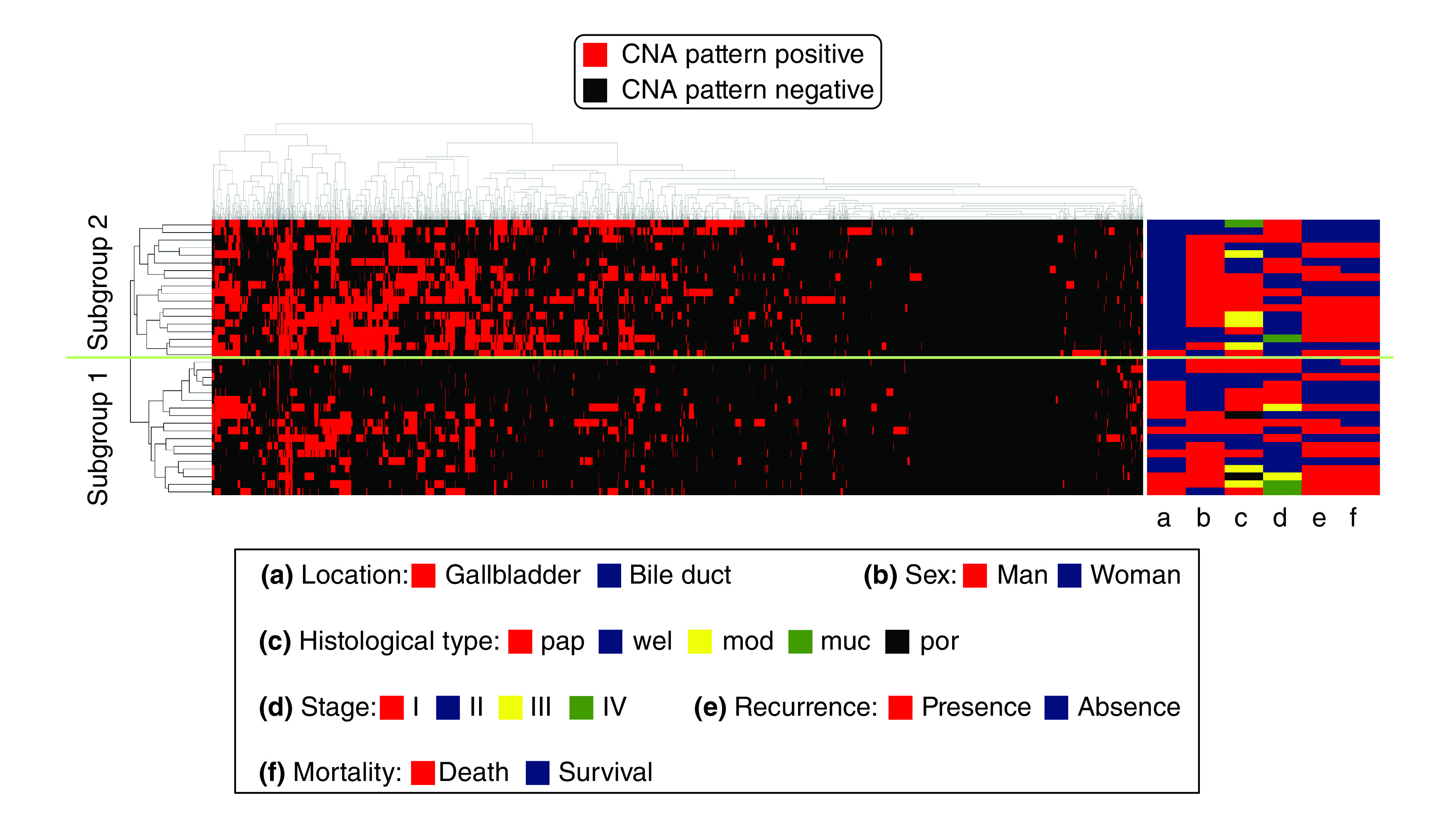
Hierarchical cluster analysis based on copy number alteration patterns in biliary tract carcinoma. There were two subgroups that were stratified by cluster analysis in biliary tract carcinoma based on SCNA patterns. SCNA: Somatic copy number alteration.

We assessed SCNA patterns using hierarchical clustering. We identified 2 distinct subgroups (subgroup 1: 18 cases; subgroup 2: 18 cases) as shown in Figure 1, in which the SCNA marker in the isolated tumor gland is indicated by the vertical line, and the horizontal lines denote ‘relatedness’ between samples.

## Clinicopathological differences between subgroups

At location, the frequency of GBC was significant higher in subgroup 1 than in subgroup 2, and there was a significant difference in the frequency of GBC between subgroups 1 and 2 (subgroup 1 >2; Table 2). However, there no significant differences in the frequency of histological type, stage, progression-free survival or overall survival between subgroups 1 and 2.

**Table 2.** Clinicopathological findings of each subgroup.

	Total (%)	Subgroup 1 (%)	Subgroup 2 (%)	p-value
Total	36	18 (50)	18 (50)	
**Sex**				0.7247
Man	24 (66.7)	11 (45.8)	13 (54.2)	
Woman	12 (33.3)	7 (58.3)	5 (41.7)	
Age (years), median (range)	70 (41–86)	70 (41–86)	70 (45–81)	0.6116
**Location**				0.0027
Gallbladder carcinoma	11	10 (90.9)	1 (9.1)	
Bile duct carcinoma	25	8 (32)	17 (68)	
Size (mm), median (range)	29 (8–123)	32.5 (15–123)	27.5 (8–75)	0.1966
Histological subtype (pap/well/mod/muc/por)	17/10/6/1/2	9/5/2/0/2	8/5/4/1/0	0.5834
**Stage**				0.5127
I	14 (38.9)	7 (50)	7 (50)	
II	17 (47.2)	7 (41.2)	10 (58.8)	
III	2 (5.6)	2 (100)	0 (0)	
IV	3 (8.3)	2 (66.7)	1 (33.3)	
**Adjuvant chemotherapy**				
Done	13 (36.1)	6 (46.2)	7 (53.8)	
None	23 (63.9)	12 (52.2)	11 (47.8)	
**Recurrence**				0.7579
Presence	21 (58.3)	10 (47.6)	11 (52.4)	
Absence	15 (41.7)	8 (53.3)	7 (46.7)	
Progression-free survival period (days), median (range)	1208.5 (97–4049)	1199 (97–4049)	1208.5 (111–3753)	
**Mortality**				0.5953
Death	18 (50)	9 (50)	9 (50)	
Survival	18 (50)	9 (50)	9 (50)	
Overall survival period (days), median (range)	1480 (151–4049)	1289 (151–4049)	1643.5 (227–3753)	

mod: Moderately differentiated adenocarcinoma; muc: Mucinous carcinoma; pap: Papillary adenocarcinoma; por: Poorly differentiated adenocarcinoma; well: Well differentiated adenocarcinoma.

### SCNAs in each subgroup

**Figure 2.**
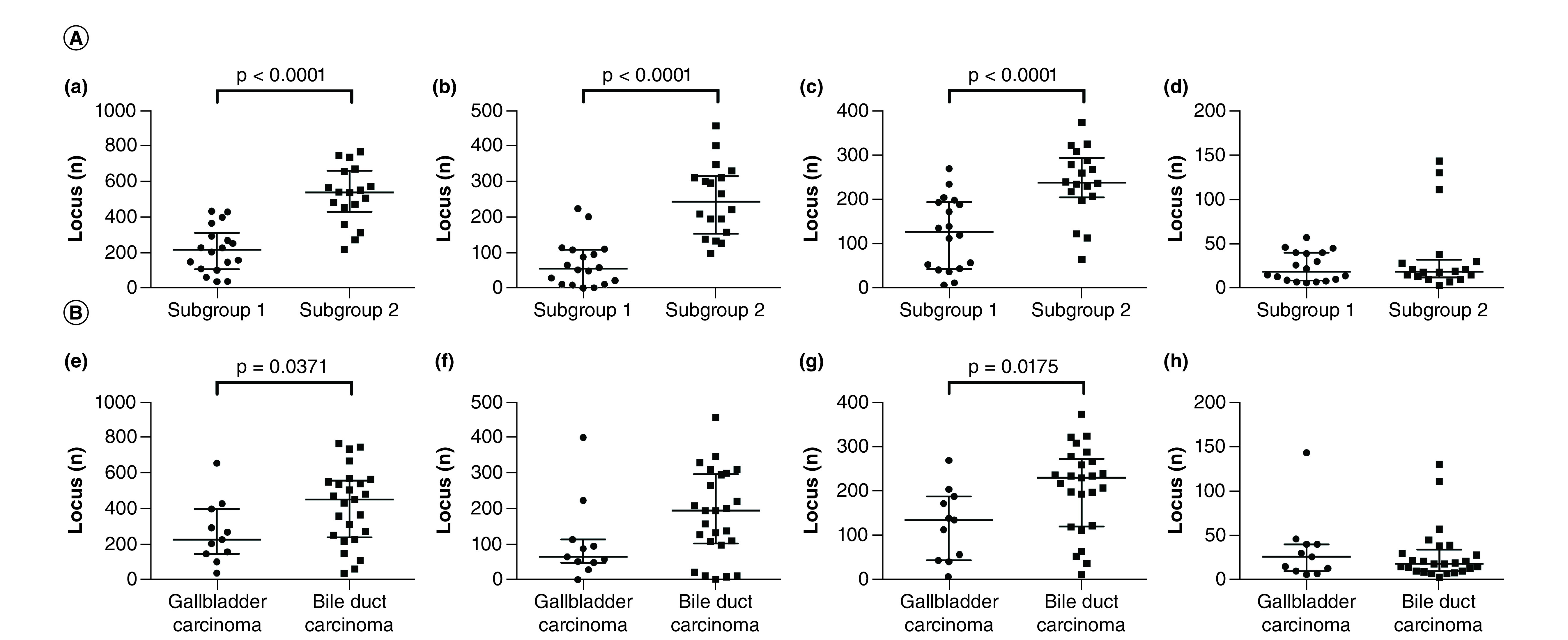
SCNAs in each subgroup. **(A)** Number of loci with somatic copy number alterations in subgroups 1 and 2. There were significant differences in the median numbers of gains and LOHs between subgroups (p < 0.0001). (a) All SCNA patterns; (b) gain pattern; (c) loss of heterozygosity pattern; (d) copy neutral loss of heterozygosity pattern; Number of loci with SCNAs in gall bladder carcinoma and bile duct carcinoma. (e) All SCNA patterns; (f) gain pattern; (g) loss of heterozygosity pattern; (h) copy neutral loss of heterozygosity pattern. LOH: Loss of heterozygosity; SCNA: Somatic copy number alteration.

The median total number of chromosomal aberrations per patient was 215.5 (range: 37–433), with a median of 55 gains (range: 1–224), 127 LOHs (range: 7–269) and 18.5 CN-LOHs (range: 6–57) in subgroup 1. In subgroup 2, the median total number of chromosomal aberrations per patient was 538.5 (range: 218–768), with a median of 243.5 gains (range: 98–457), 237.5 LOHs (range: 64–373) and 18.5 CN-LOHs (range: 3–143). There were significant differences in the total numbers of SCNAs between subgroups 1 and 2 (p < 0.0001). Moreover, significant differences were observed in the median numbers of gains and LOHs between subgroups (p < 0.0001). The association is shown in Figure 2.

Regions of gains detected in more than 40% of cases were located at 14q32.33, 18p11.32–p11.21, 17q11.2–q12 and 1q21.1–q31.3 (in decreasing order of frequency) in subgroup 1 (more than 40% of cases); however, there were many cases showing regions of gains detected in more than 40% of cases in subgroup 2. The regions are listed in Supplementary Table 1. LOHs detected in more than 40% of cases occurring in subgroups 1 and 2 are summarized in Supplementary Table 1. Moreover, CN-LOHs (more than 30% of cases) were found at 1p33–p32.3 in subgroup 1. No CN-LOHs (more than 30% of cases) were found in subgroup 2. These results are summarized in Supplementary Table 1.

### Differences in SCNAs between subgroups

We examined differences in SCNAs between subgroups 1 and 2. Regions of SCNAs detected in more than 40% of cases (CN-LOH detected in more than 30% of cases) were selected for comparisons among subgroups. Significant differences in gains between subgroups 1 and 2 were found at 1p, 1q, 2p, 2q, 3q, 7p, 7q, 8p, 8q, 11q, 17q, 19q and 20q (subgroup 2 >subgroup 1; Table 3). Significant differences in LOHs between subgroups 1 and 2 were found at 4q, 9p, 12q, 14q, 17p, 18q and 19p (subgroup 2 >subgroup 1; Table 3). No significant differences in CN-LOH were observed between subgroups 1 and 2.

**Table 3.** Significant differences in the frequencies of copy number alterations between subgroup 1 and 2.

Chromosomal regions	Subgroup 1 n = 18 (%)	Subgroup 2 n = 18 (%)	p-value^†^
**Gain**			
7p22.3–p11.1	1–3 (5.6–16.7)	11–15 (61.1–83.3)	<0.01
7q11.21–q22.1	1–2 (5.6–11.1)	10–14 (55.6–77.8)	<0.01
3q26.31–q26.32	2–3 (11.1–16.7)	11–13 (61.1–72.2)	<0.01
20q11.21–q13.33	0–2 (0–11.1)	10–13 (55.6–72.2)	<0.01
3q23–q25.33	1–2 (5.6–11.1)	9–12 (50–66.7)	<0.01
3q27.1, 3q29	2 (11.1)	11 (61.1)	<0.01
2q31.1–q32.3	1–2 (5.6–11.1)	10–11 (55.6–61.1)	<0.01
2q22.1–q24.2	0–1 (0–5.6)	8–11 (44.4–61.1)	<0.01
11q12.2–q14.3	0–1 (0–5.6)	7–11 (38.9–61.1)	<0.01
2q33.2, 2q34 7q31.2–q31.33	1 (5.6)	10 (55.6)	<0.01
1p12, 2q12.1, 2q14.2 2q36.3	1 (5.6)	9 (50)	<0.01
2p23.1–p16.2, 11q24.2–q25	0 (0)	7–9 (38.9–50)	<0.01
2p24.3–p23.3	0 (0)	7–8 (38.9–44.4)	<0.01
8q21.3–q24.22	4–7 (22.2–38.9)	11–15 (61.1–83.3)	<0.05
1q24.1–q24.2	6 (33.3)	13 (72.2)	<0.05
3q26.1–q26.2	3 (16.7)	12 (66.7)	<0.05
17q21.1	4 (22.2)	12 (66.7)	<0.05
17q22	5 (27.8)	12 (66.7)	<0.05
8q11.1–q12.3	4 (22.2)	11–12 (61.1–66.7)	<0.05
1q32.2, 8p11.22, 8p11.1 8q21.11	4 (22.2)	11 (61.1)	<0.05
1q42.11–q44, 3q27.2–q28	3 (16.7)	10–11 (55.6–61.1)	<0.05
2q24.3, 2q33.1, 2q33.3 3q26.33, 7q22.2–q31.1	2 (11.1)	10 (55.6)	<0.05
19q13.31–q13.32, 19q13.41	3 (16.7)	10 (55.6)	<0.05
7q32.1–q34	1–3 (5.6–16.7)	8–10 (44.4–55.6)	<0.05
1p36.23–p36.22, 2q37.1 19q13.43	2 (11.1)	9 (50)	<0.05
2p16.1–p14	1–2 (5.6–11.1)	8–9 (44.4–50)	<0.05
2q12.2–q14.1, 2q35	1 (5.6)	8 (44.4)	<0.05
2p25.3–p25.1	1 (5.6)	7–8 (38.9–44.4)	<0.05
**CNLOH**			
None			
**LOH**			
18q22.1–q22.2	7 (38.9)	16 (88.9)	<0.01
19p13.3–p13.2	3–4 (16.7–22.2)	15 (83.3)	<0.01
12q21.1–q24.32	0 (0)	7–12 (38.9–66.7)	<0.01
4q13.1	1 (5.6)	9 (50)	<0.01
17p13.3–p13.2	10–11 (55.6–61.1)	17 (94.4)	<0.05
17p12	11 (61.1)	17 (94.4)	<0.05
9p24.2, 9p21.2	9 (50)	16 (88.9)	<0.05
18q12.2–q21.33	7–8 (38.9–44.4)	15–16 (83.3–88.9)	<0.05
9p13.3–p13.2	6–8 (33.3–44.4)	14–16 (77.8–88.9)	<0.05
14q31.3–q32.33	4 (22.2)	11–12 (61.1–66.7)	<0.05
19p13.13–p13.12	3–4 (16.7–22.2)	11–12 (61.1–66.7)	<0.05
14q23.1, 17p11.1	3 (16.7)	11 (61.1)	<0.05
14q23.3–q24.2, 14q31.1	4 (22.2)	11 (61.1)	<0.05
14q11.2–q12	2–3 (11.1–16.7)	10–11 (55.6–61.1)	<0.05
14q13.2, 14q21.2	3 (16.7)	10 (55.6)	<0.05
12q24.33	1 (5.6)	8 (44.4)	<0.05

† Benjamini–Hochberg false discovery rate adjusted p-value.

CNA: Copy number alteration; CNLOH: Copy-neutral loss of heterozygosity; LOH: Loss of heterozygosity.

### Overall survival & disease-free survival in the stratified subgroups

The proportions of progression-free cases and mortalities were 41.7% (15/36 BTCs) and 50% (18/36 BTCs), respectively (Table 1). In addition, Kaplan–Meier analysis was performed to determine and compare progression-free survival and overall survival according to each stratified SCNA pattern (subgroups 1 and 2). However, there were no significant differences in progression-free and overall survival between subgroups 1 and 2.

### SCNAs in GBC & BDC

The median total number of chromosomal aberrations per patient was 227 (range: 38–657), with a median of 65 gains (range: 1–401), 135 LOHs (range: 7–269) and 26 CN-LOHs (range: 6–143) in GBC. In BDC, the median total number of chromosomal aberrations per patient was 453 (range: 37–768), with a median of 195 gains (range: 2–457), 230 LOHs (range: 12–373) and 18 CN-LOHs (range: 3–130). There were significant differences in the total numbers of SCNAs between GBC and BDC (p = 0.0371). Moreover, significant differences were observed in the median numbers of LOHs between GBC and BDC (p = 0.0175). Finally, gains and CN-LOHs were common between GBC and BDC.

Regions of gains detected in more than 40% of cases were located at 14q32.33, 8q11.1–q24.3, 17q11.2–q12 and 1q21.1–q44 (in decreasing order of frequency) in GBC. In contrast, regions of gains detected in more than 40% of cases were located at 7q11.21–q36.3, 7p22.3–p11.1, 17q11.2–q25.3, 8q11.1–q24.3, 18p11.32–p11.21, 1q21.1–q44, 3q22.3–q29 and 18q11.1 in BDC. LOHs detected in more than 40% of cases were found at 4q13.2, 9p24.3–p13.1, 17p13.3–p11.2, 3p21.31, 5q12.2–q35.3 and 14q24.3 in GBC and at 17p13.3-p11.1, 18q11.2–q23, 14q11.2–q32.33, 9p24.3–p13.1, 6q12–q27, 19p13.3–p12, 5q11.1–q35.3, 1p36.33–p35.1 and 4q12–q35.2 in BDC. Moreover, CN-LOHs (more than 30% of cases) were found at 1p33–p32.3 and 17q23.2 in GBC and at 1p32.3 in BDC, respectively. These results are summarized in Supplementary Table 2.

### Differences in SCNAs between lesion types

Next, we examined differences in SCNAs between the two lesion types. Regions of SCNAs detected in more than 40% of cases were selected for comparison. Significant differences in gains between GBC and BDC were found at 7q11.21–q31.33, 7p11.1, 7p13–p12.2, and 2q31.3 (BDC > GBC; Table 4). No significant differences in LOH or CN-LOH were observed between GBC and BDC.

**Table 4.** Significant differences in the frequencies of somatic copy number alterations between gallbladder carcinoma and bile duct carcinoma.

Chromosomal regions	Gallbladder carcinoma n = 11 (%)	Bile duct carcinoma n = 25 (%)	p-value^†^
**Gain**			
7q11.21–q31.33	0 (0)	11–16 (44–64)	<0.05
7p13–p12.2	0 (0)	12–14 (48–56)	<0.05
7p11.1	0 (0)	12 (48)	<0.05
2q31.3	0 (0)	11 (44)	<0.05
**CNLOH**			
None			
**LOH**			
None			

† Benjamini–Hochberg false discovery rate adjusted p-value.

CNA: Copy number alteration; CNLOH: Copy-neutral loss of heterozygosity; LOH: Loss of heterozygosity.

## Discussion

In previous studies, two complementary methods, in other words, karyotyping and comparative genomic hybridization (CGH), were used to examine chromosomal aberrations in BTCs [9,11]. However, in recent works, the SNP array method is preferred for identification of SCNAs. The difference between CGH and SNP arrays is that CN-LOH can be detected in SNP arrays [21]. CN-LOH is defined as LOH by duplication of a maternal or paternal chromosome or chromosomal region and concurrent loss of the other allele [21,22]. This definition suggests that the effect of a mutated allele with CN-LOH may be enhanced twofold [21–23]. Therefore, cancer cells may have a growth advantage if the copy number in a chromosomal region is preserved [21–23]. CN-LOH is a frequent chromosomal alteration in human hematological malignancies, such as leukemia, mantle cell lymphoma and follicular lymphoma [21,23]. For cases of hematological malignancy, samples can be enriched in tumor cells by flow cytometry, making this type of analysis suitable for these diseases [21]. Isolated cancer glands enable us to detect CN-LOH in solid tumors, which may otherwise be underestimated owing to contamination of interstitial cells without CN-LOH [15]. In the current study, CN-LOH at 1p33–p32.3, 17q23.2 and 1p32.3 was detected in both GBC and BDC. Although these loci with CN-LOH may contribute to development of BTC, the role of CN-LOH in molecular carcinogenesis of BTC remains unknown. However, we hypothesize that CN-LOH may give rise to homozygosity in a mutated tumor-suppressor gene, thereby promoting tumor growth or chemotherapy resistance; studies of this topic may facilitate elucidation of the role of CN-LOH [22].

We performed cluster analysis to avoid arbitrary segregation in BTC including GBC and BDC we examined based on SCNA pattern. This approach is necessary to identify molecular carcinogenesis objectively before the difference in the molecular alterations between GBC and BDC is evaluated. According to this principle, we divided two step analyses into cluster analysis of BTC and individual analysis for each lesion of GBC and BDC. According to this theory, in the current study, we stratified BTCs into two subgroups: subgroups 1 and 2. Subgroup 1 was characterized by a low frequency of SCNAs compared with that of subgroup 2, whereas subgroup 2 was closely associated with a high frequency of SCNA. In the current study, subgroup 1 was assigned to GBC, and subgroup 2 was assigned to BDC. Accordingly, despite belonging to the same category of BTCs, molecular alterations in BDC were quite different from those in GBC in terms of SCNA patterns. In addition, this finding is supported by the observation that the total number of SCNAs was significantly higher in BDC than in GBC. This result may have novelty to evaluate carcinogenesis of BTCs, given that comprehensive genomic profiling has a developing role in guiding systemic, precision anticancer therapy.

Previous studies have shown that chromosomal loci at 1p, 3p, 6q, 6p, 8p, 10q, 12q, 12p, 17p, 18q and 22q are frequently deleted in BTCs, including gall bladder cancer and intra- and extrahepatic hepatocellular carcinomas [9–13]. By contrast, chromosomal loci at 1q, 2pq, 7p, 8q, 11q, 12p, 13q, 17q, 19q, 20q and chromosomes 17 and 20 are commonly gained (amplified) in BTCs [9–13]. In the current study, copy number gains at 14q, 8q, 17q and 1q were frequently found in GBC, whereas those at 7q, 17q, 8q, 18q, 1q and 3q were frequent genetic events in BDC. Furthermore, LOHs were frequent on 4q, 9p, 17p, 3p, 5q, 14q, 4q, 6q, 10q and 18q in GBC and at 17p, 18q, 14q, 9p, 6q, 19p, 5q, 1p and 4q in BDC. Although different results regarding SCNAs in BTC have been reported, this discrepancy may depend on sampling (crypt isolation vs fresh sample, which may be contaminated with interstitial cells), analytical platform (CGH vs SNP array) and ethnic differences [20]. In the current study, our results appeared to be reliable, with reproducible SCNA data obtained specifically from neoplastic cells, without interstitial cell contamination.

In the current study, gain at 14q32.33 and LOH at 4q13.2 were the most frequent events in GBC. *AKT1*, which is located at 14q32.33, is an important oncogene and component of the phosphatidylinositol 3-kinase/AKT1/mTOR pathway, which is closely associated with many types of human carcinogenesis [24]. Wencong *et al.* showed that FOXK1 promotes the proliferation and metastasis of GBC by activating the AKT/mTOR signaling pathway [25], supporting the findings of our study. Additionally, a previous study revealed that although LOH on 4q13.2 is frequently found in hepatocellular carcinoma, this molecular alteration has been reported even less frequently [26]. Notably, LOH on 4q13 was also found in cervical cancer and malignant lymphoma [27,28]. According to this finding, LOH at 4q13 may play crucial roles in the carcinogenesis of GBC. *RASSF6* (Ras-association domain family 6), located at 4q13.3, encodes a member of the *RRASSF* family and may be a candidate gene [29]; however, no studies have reported the role of this gene in BTC. Members of this family form the core of a highly conserved tumor-suppressor network, the Salvador-Warts-Hippo pathway, which is a significant regulator of growth, tissue regeneration and stem cell pluripotency [30]. The functional role of this gene is as a tumor-suppressor gene, with roles in cancer metastasis.

Previous studies have shown that accumulation of SCNAs is correlated with patient outcomes [13–15]. In the current study, however, no associations of disease-free survival or overall survival with SCNAs were observed. Accumulation of SCNAs during neoplastic progression may be necessary for tumor growth and metastatic potential in many cancers [7,13]. However, various factors, including genetic mutations, DNA methylation, histological type and stage, may be involved in determination of patient survival [7,13]. An integrated approach to prediction of patient prognosis may be needed in the near future.

Previous studies have shown that there are differences in the clinicopathological findings and embryological development of intrahepatic and extrahepatic BDCs [31,32]. However, it is unclear whether molecular alterations in intrahepatic BDC are different from those in extrahepatic BDC. These two types of cancer exhibit similar pathological findings, including histological features and some genetic alterations [31,32]. According to this finding, we assigned both of these cancers to the same category in the current study.

There were some limitations to this study. First, the number of cases evaluated in our study might be small. Large studies, such as genome-wide studies in The Cancer Genome Atlas, provide useful information for evaluation of the carcinogenesis of BTC [6]. However, we believe that the current study, which used isolated tumor glands, provided novel insights into the molecular mechanisms of human BTC, because it is essential to obtain correct target cells for accurate evaluation of genetic alterations. In addition, This is the first study to examine SCNAs occurring in BTC, including GBC and CCC (BDC) using a crypt (gland) isolation method. Second, we could not validate the findings of the current study because BTC is an infrequent type of human neoplasia. However, it may be possible to integrate publicly available data to determine whether the clusters identified in this study have been replicated in other studies. In the current study, however, we used a different platform than in other studies; therefore, it may be difficult to examine reproducibility using an open-source database. In addition, isolated tumor glands, which were obtained as pure tumor glands without stromal cells, were used in the current study, affecting the results obtained for SCNA patterns in cancer cells.

## Conclusion

We examine SCNAs in BTC, including GBC and BDC, using isolated tumor glands without contamination of interstitial cells. We found multiple copy number gains and LOHs in both GBC and BDC. In addition, we detected CN-LOH, which may be a rare genetic event in BTC. Finally, our findings suggested that there were two subgroups in BTC, including subgroup 1 (molecular alterations in GBC) and subgroup 2 (molecular alterations in BDC). Overall, these findings provide insights into evaluation of the molecular carcinogenesis of BTC. Further studies are needed to expand upon and confirm our results in the near future.

**Summary points**

- We aimed to identify the roles of somatic copy number alterations (SCNAs) in biliary tract carcinoma, including gallbladder carcinoma (GBC) and biliary duct carcinoma (BDC), using an single nucleotide polymorphism array.
- In hierarchical cluster analysis, two clusters were identified, subgroup 1 (low SCNA frequency) and subgroup 2 (high SCNA frequency).
- GBC was predominant in subgroup 1, whereas BDC was predominant in subgroup 2.
- GBC and BDC may have different genetic backgrounds in terms of SCNAs.
- These findings may be helpful for establishing the molecular mechanisms mediating the carcinogenesis of biliary tract carcinomas.
